# Impact of Surgical Stabilization of Flail Chest Injuries on Postoperative Computed Tomography Lung Volumes

**DOI:** 10.3390/jcm14113644

**Published:** 2025-05-22

**Authors:** Moses K. D. El Kayali, Georg Böning, Moritz Günther Mewes, Karl F. Braun, Karin Steinecke, Konrad Neumann, Ulrich Stöckle, Vera Jaecker, Marcel Niemann

**Affiliations:** 1 Center for Musculoskeletal Surgery, Charité—Universitätsmedizin Berlin, Corporate Member of Freie Universität Berlin and Humboldt-Universität zu Berlin, Augustenburger Platz 1, 13353 Berlin, Germany; 2 Department of Radiology, Charité—Universitätsmedizin Berlin, Corporate Member of Freie Universität Berlin and Humboldt-Universität zu Berlin, Augustenburger Platz 1, 13353 Berlin, Germany; 3 Department of Trauma Surgery, University Hospital Rechts der Isar, Technical University of Munich, 81675 Munich, Germany; 4 Department of Anaesthesiology and Intensive Care Medicine, Charité—Universitätsmedizin Berlin, Corporate Member of Freie Universität Berlin and Humboldt-Universität zu Berlin, Augustenburger Platz 1, 13353 Berlin, Germany; 5 Institute of Biometry and Clinical Epidemiology, Charité—Universitätsmedizin Berlin, Corporate Member of Freie Universität Berlin and Humboldt-Universität zu Berlin, Augustenburger Platz 1, 13353 Berlin, Germany; 6 Julius Wolff Institute, Berlin Institute of Health at Charité—Universitätsmedizin Berlin, Augustenburger Platz 1, 13353 Berlin, Germany

**Keywords:** multiple trauma, flail chest, unstable thorax, osteosynthesis, thorax stabilization

## Abstract

**Background:** Flail chest (FC) injuries are multiple adjacent segmental rib fractures, commonly associated with a high complication and mortality risk. Recent evidence suggests that the early surgical stabilization of FC injuries is beneficial for restoring breathing mechanics. However, little is known about the effects on lung volumes when invasive ventilation is performed after surgery. **Methods:** This retrospective study included multiple trauma (MT) patients operatively treated for an FC injury between 2011 and 2024. The indication for surgery was based on a computed tomography (CT) proof of an FC, objectifiable paradoxical breathing, and prolonged weaning. All patients treated used a single osteosynthesis system. Lung volumes were manually measured in preoperative and postoperative CT scans of the thorax in the thinnest CT reconstructions available. The primary outcomes of interest were the changes in the lung volumes following surgical stabilization of the FC. **Results:** During this study, 21 patients (90.48% male) were operatively treated for their FC injury. All patients had been affected by high-energy trauma. The corresponding median Injury Severity Score (ISS) was 26 (IQR 17.5, 33). Patients suffered 7 (IQR 6, 10) and 6 (IQR 2, 9) fractured ribs of the left and right hemithorax, respectively. Three (IQR 0, 3) and two (IQR 0, 3) ribs of the left and right hemithorax, respectively, were stabilized at 7 (IQR 2, 18) days post admission. There were no significant changes in the lung volumes comparing preoperative and postoperative CT scans. **Conclusions:** As this study did not detect CT volume changes comparing preoperative and postoperative scans, CT scans following surgery may not qualify for an objective measurement of the surgical effectiveness regarding lung volume restoration in the short-term follow-up. Long-term changes in CT-measured lung volume changes need to be evaluated to prove an objective surrogate parameter for surgical effectiveness regarding the restoration of the thorax integrity.

## 1. Introduction

Almost 10% of the global population dies annually due to severe trauma [1], which is simultaneously the most probable cause of death in adults younger than 45 [2]. These patients commonly suffer from severe injuries to the thorax [3], which are believed to cause up to 25% of the overall trauma-associated mortality [4]. In fact, up to 1/3 of all patients admitted to the ward following trauma suffer from thorax injuries [5], which is even higher in multiple-injured patients [3]. In 2015, the National Trauma Data Bank, the largest aggregation of US trauma data, reported 9.5% of all thorax-related injuries to be severe according to the abbreviated injury scale (AIS ≥ 3) [6].

The most common thorax injuries are rib fractures [7,8]. These fractures affect up to 10% of all patients admitted following blunt trauma [9,10]. Flail chest (FC) injuries are a particular type of rib fracture, defined as at least three adjacent segmental fractures [11]. Only 7% of all rib injuries fulfill the criteria of FC injuries [12]. According to the Traumaregister DGU^®^, FC injuries affected over 12,000 patients between 2009 and 2017, corresponding to over 1300 annual cases in Germany [13]. This is highly comparable to US data from the National Trauma Data Bank from 2007 to 2009, which registered almost 3500 patients suffering from FC injuries, corresponding to 1155 FC cases per year [14]. In the Netherlands, 573 patients with FC injuries were reported to the national trauma register between 2015 and 2017, corresponding to 191 FC injuries per year [15]. These injuries are associated with an increased risk for paradoxical chest wall movements due to the mechanical decoupling of the fractured segment [11]. Osseous injuries of the thorax, especially FC injuries, are an independent risk factor for respiratory infections, prolonged invasive ventilation, and mortality [16,17,18].

Traditionally, most FC injuries are treated non-operatively. Back in the day, treatment concepts on FC injuries consisted of internal pneumatic stabilization of the injuries [19]. Even earlier, patients were strategically intubated and invasively ventilated for pre-determined periods to facilitate healing through the internal stabilization resulting from the pressure-ventilation. Modern treatment concepts encompass a multidisciplinary approach including sufficient analgesia, respiratory support as needed, breathing exercises, and physiotherapy [20,21]. However, recent evidence suggests that the superiority of early operative treatment is evident in terms of duration of invasive ventilation, length of stay (LOS), and mortality [18,22,23,24,25,26,27,28,29,30], which led to an increase in operated FC patients up to 16% [31]. However, surgery needs to be scheduled at the earliest possible time following the injury, optimally within 72 h, to achieve the highest patient benefits [18]. This may be challenging in severely injured patients, as they are regularly not sufficiently stable within this short period. This commonly results in an exclusion of these patients from most prospective studies [32,33,34,35], ultimately leading to a lack of evidence regarding the surgical strategy for multiple-injured patients with FC injuries. Furthermore, potential risks associated with surgery need to be considered and evaluated in conjunction with the previously mentioned conservative treatment. The overall risk of surgical FC stabilization is approximately 13% [36]. This includes wound healing disorders [36], local bleeding [37], and implant-related pain, resulting in an implant removal rate of up to 50% [38].

Modern implants comprise intramedullary splints [39], angular stable titanium plates [39], or claw-shaped titanium plates [40,41]. These implants allow for an anatomic reduction and biomechanically stable fracture fixation, thereby simultaneously restoring chest wall stability. Despite the many questions that have been answered, various questions remain open regarding the optimal surgical strategy when managing FC injuries. The Chest Wall Injury Society is the most critical specialist group leading the field of evidence and recommendations for managing thorax injuries, including FC injuries. Their regularly updated guidelines help clinicians choose the most optimal strategy for handling FC patients [42].

Computed tomography is essential for the initial and follow-up diagnosis of thoracic injuries. It has been proven to be superior to conventional X-rays with higher odds of detecting intrathoracic injuries [43]. Due to its broad availability and rapid protocol, CT is the gold diagnostic standard, especially in severely injured patients [44]. Various protocols have been developed to shorten scans and image reconstruction while maintaining sufficient global imaging [45]. Furthermore, it is the only widely available imaging modality that enables three-dimensional imaging, facilitating adequate postoperative control following FC stabilization. Therefore, postoperative CT scans are commonly used in routine follow-up [46]. Recently, a few studies suggested a significant increase in CT lung volumes following the operative stabilization of FC injuries [46,47,48], indicating that CT scans may serve as a surrogate parameter for evaluating thorax reconstruction effectiveness. However, these studies focused on the outpatient follow-up several months after the operative treatment. The initial impact of FC stabilization on the lung volumes and the invasiveness of the mechanical ventilation have, thus far, not been assessed.

This study aims to assess early CT-based lung volume changes in operatively treated FC patients before and after surgery and to compare the changes in respiratory invasiveness after stabilization of the flail segments. With this, early postoperative CT scans shall be evaluated as a surrogate parameter for reconstruction effectiveness.

## 2. Methods

The local ethics committee approved the study protocol, and this study was conducted per the Declaration of Helsinki.

We conducted a retrospective study between September 2011 and January 2024 at a level 1 trauma center. All patients surgically treated for FC at the study center during the abovementioned study period were eligible for inclusion. FC was defined as (a) at least three contiguous ribs with segmental fractures or (b) more than five adjacent rib fractures with a resulting paradoxical breathing pattern.

The electronic medical data system SAP (SAP ERP 6.0 EHP4, SAP AG, Walldorf, Germany) was searched for eligible patients using respective procedural coding (Operationen und Prozedurenschlüssel [OPS]). In Germany, these codes are the basis for billing medical procedures. The surgical stabilization of FC injuries using an open approach is the standard approach at the study center. Since 2019, these procedures have been coded as 5-346.c* (open unilateral surgical stabilization of the thorax) and 5-346.d* (open bilateral surgical stabilization of the thorax). Before 2019, 5-346.5 had primarily been used. Therefore, we decided to conduct a systematic search for 5-346.*, which was selected by our department as the primary provider of the procedure. This allowed us to select every thoracic surgical intervention performed by our department. Of these patients, the surgical protocols were manually reviewed for study inclusion.

The indication for the surgical stabilization of FC injuries is typically determined at the study center using a multidisciplinary approach, which has been previously described [49] and is based on the international guideline recommendations of the Chest Wall Injury Society [42]. In short, surgical stabilization is considered in patients with (a) imaging-based proof of an FC injury, (b) a paradoxical breathing pattern, and (c) a prolonged weaning process. The latter is commonly defined as a failed attempt requiring a re-intubation within 48 h after extubation. At our clinic, the MatrixRib^TM^ system (DePuy Synthes, Raynham, MA, USA) is the sole system used to stabilize FC injuries, and the standard surgical treatment concept employed has been described before [49].

We assessed demographic, injury, and procedural-related data in patients eligible for study inclusion. Demographics included sex, age, and pre-existing medical conditions represented by the Charlson Comorbidity Index (CCI) and the American Society of Anesthesiologists (ASA) risk classification. Further, we assessed ICU and hospital length of stay (LOS) and injury severity. The latter included the highest AIS per body region, the corresponding Injury Severity Score (ISS), associated injuries, and in-hospital mortality. The Injury Severity Score (ISS) was calculated as previously described [50]. Additionally, ventilation data, which included peak inspiratory pressure (P_IP_), positive end-expiratory pressure (PEEP), the fraction of inspired oxygen (FiO_2_), tidal volume (TV), respiratory minute ventilation (RMV), and respiratory rate (RR), were recorded. These markers are documented hourly and automated in the ICU’s electronic medical data system COPRA (COPRA 6/COPRA 5, COPRA System GmbH, Berlin, Germany). We also assessed the duration of mechanical and non-invasive ventilation, the need for re-intubation after previous extubation, and the tracheotomy rate. Procedural data included the number of stabilized ribs and surgical revisions in the follow-up.

Between 2011 and 2024, 21 patients were operated on at the study center due to an FC. All patients included in this study were multiple-injury patients following high-impact trauma. The patients were 49 (IQR 38, 68) years of age, and most (90.48%) were male. The CCI was 1 (IQR 0, 3), and the ASA risk classification was 3 (IQR 2, 4). There was no systematic documentation of weight in this cohort. Despite data of four patients (19.05%) being missing, patients had a median height of 180 cm (IQR 173, 190), a weight of 79 kg (IQR 70, 90), and a BMI of 23.37 (IQR 21.81, 27.69). Besides one missing report, 18 patients (85.71%) had no previous chest or lung disease, while 2 (9.52%) did. One patient already had chronic obstructive pulmonary disease; the other one had asthma. The ISS of the cohort was 26 (IQR 18, 33), and upon hemodynamic stabilization, all patients were transferred to the intensive care unit (ICU). Most of these patients (80.95%) required mechanical ventilation at some point, which was performed for 6 days (IQR 2, 11). We aimed to end mechanical ventilation as early as possible and immediately began non-invasive ventilation (NIV) as required in 86.67% of these patients. NIV was continued for 7 (IQR 5, 8) days. During that period, 15.79% of patients needed re-intubation due to respiratory failure. Ultimately, 28.57% of the included patients required a tracheotomy during their stay on the ward due to persisting ventilator dependency. The overall ICU LOS was 22 (IQR 9, 33) days. Following ICU care, patients were transferred directly to a hospital or rehabilitative clinic providing continued care or to the regular ward for a total stay of 26 (IQR 20, 36) days. Another 28.57% were discharged home, 19.05% to an ICU, 28.57% to a regular ward rehabilitative clinic, 9.52% to a psychiatric hospital, and 4.67% to a nursing home. Intrahospital mortality was 9.52%.

**Figure 1:**
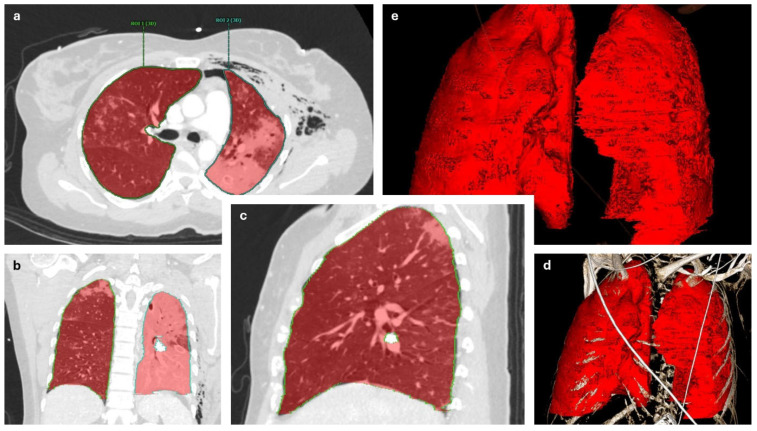
Manual measurement of lung volumes. (**a**) Axial, (**b**) coronal, (**c**) sagittal, (**d**) volume rendering (VR), (**e**) VR without bones. ROI: Region of interest.

We measured lung volumes on preoperative and postoperative computed tomography (CT) images to assess the volumetric effect of the surgical stabilization of the FC injuries. In the thinnest axial CT reconstructions available, volumes were digitally measured in Visage^®^ (Visage^®^ for Microsoft Windows, Version 7.1, Visage Imaging, Inc., Pro Medicus Limited, Richmond, Australia). Measurements were performed in the earliest CT scans conducted after hospital admission and postoperative CT scans conducted during the hospital stay. CT examinations were performed according to our clinical standards, predominantly on the identical multi-slice scanner (GE Revolution HD, GE Healthcare, Chicago, IL, USA). For all examinations, multiplanar reconstructions with a slice thickness of less than 1 mm were reconstructed in different kernels using iterative algorithms. Lung volumes were measured from the airway to the end of each lung hilum by outlining the pulmonary tissue. This included areas with contusions to detect the effect of thoracic wall reconstruction most accurately. Pneumothoraces and hemothoraces were not included in these volumetric measurements. All measurements were performed independently by two authors (M.E., M.N.), who were supervised by a third author (G.B.), and all calculations are based on the means of these measurements. Additionally, apex–diaphragm distances were measured from the apex pulmonis to the tip of the diaphragm at the position of the third bronchial branching of both sides on the CT images mentioned above (Figure 1).

The statistical analysis used GraphPad Prism (GraphPad Prism 10 for Mac OS, Version 10.2.3, GraphPad Holdings, LLC, San Diego, CA, USA). The primary outcomes of interest were the changes in the lung volumes following surgical stabilization of the FC. In dependent samples, continuous variables were assessed using the Wilcoxon signed-rank test.

We used SPSS (SPSS Statistics for Windows, Version 29, IBM Corp., Armonk, NY, USA) for multiple imputations (MIs) of missing values in patients who had not received a CT following surgery. This was necessary, as 6 out of the 21 included patients had not received any postoperative CT scan at our clinic. We decided on MI to prevent further data loss regarding the mentioned endpoints. MI is a statistical method addressing missing data by replacing each missing value with multiple plausible values. Plausibility is derived from the relations among the remaining data [51]. We generated n = 5 imputation samples using the variables pre- and postoperative breathing modus, lung volume, and apex–diaphragm distance for plausibly predicting the missing values. This generates an MI-marked dataset including the original data and five requested imputation samples. Afterwards, SPSS allows for a pooled analysis of the MI, which was performed using dependent *t*-tests. The outcome measures for these tests were the pre- and postoperative lung volumes. The pooled analysis tests each dataset separately before combining them for the overall analysis. However, the pooled analysis is only considered for MI testing.

Unless stated otherwise, discrete and continuous variables are represented as the median and interquartile range (IQR), and categorical variables are represented as frequencies and portions of a whole [%]. In the pooled analysis of the MI, data are presented as the mean with standard error of the mean [SEM]. All tests are two-tailed, and in this observational study, all *p*-values are considered exploratory.

## 3. Results

### 3.1. Injury Characteristics

Patients suffered 7 (IQR 6, 10) and 6 (IQR 2, 9) fractured ribs of the left and right hemithorax, respectively. Of these, 61.90% and 33.33% were FC injuries on the left and right sides, respectively, while 28.57% of the patients had bilateral FC injuries. Moreover, 46.15% of the patients had lung contusions, of which 35.71% were bilateral. Three lobes (IQR 1, 3) per patient were rated as contused based on imaging. Furthermore, 19.05% of the patients had concomitant fractures of the sternum, 42.86% ipsilateral fractures of the clavicle, and 33.33% ipsilateral fractures of the scapula. None of the scapula fractures needed osteosynthesis, but 33.33% of the clavicle fractures did.

Three of the patients had additional intrathoracic injuries. One patient suffered from a traumatic cardiac dislocation, which we previously reported [52], and an aortic dissection type Stanford A. Another patient also suffered from a type A dissection, and the last one suffered from a ruptured truncus pulmonalis on the side of the FC injury. The aortic dissections were treated with thoracic endovascular aortic repair, and the ruptured truncus was directly sutured. All of these severe intrathoracic injuries were operatively treated on the day of hospital admission.

No patients had associated cervical spine injuries, but six (28.57%) had thoracic and five (23.81%) had lumbar spine injuries. According to the AO Spine thoracolumbar spine injury classification system [53], these included one A1, one A2, two B2, one B3, and one type C fracture. While the A1 fracture was conservatively treated, the B2 fractures were treated with open surgery, and the other fractures were treated with percutaneous spinal fusion. Surgeries were performed at 3 days (IQR 1, 5) after hospital admission and 3 days (IQR 2, 23) before surgical stabilization of the FC injuries. Further, three patients suffered from A1 and one from a type C fracture of the lumbar spine. The latter one was treated via open spinal fusion on the day of hospital admission, which was 9 days before the surgical stabilization of this patient’s FC injury. Lastly, six patients (28.57%) suffered from associated limb injuries, including one patient with a proximal humerus, bilateral tibial head, and an unimalleolar ankle fracture; one patient with a distal humerus and olecranon fracture, and a ligament injury of one knee; and two patients with bimalleolar fractures. Limb injuries were fixed 3 days (IQR 1, 8) after hospital admission and 3 days (IQR 0, 10) before surgical stabilization of the FC injuries.

**Figure 2:**
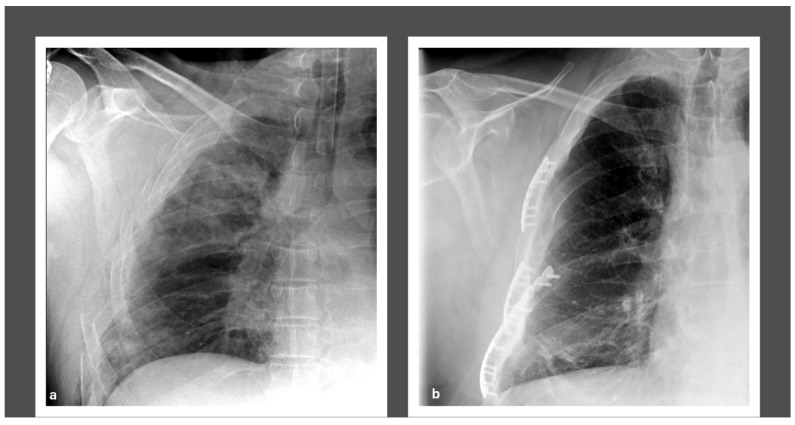
Plain radiographs before (**a**) and after (**b**) surgical stabilization of a flail chest injury.

All patients initially required a thorax drain for 12 (IQR 8, 19) days. None of the patients required a secondary drain after the removal, due to persisting or recurrent pneumothorax. However, one patient was operated on after the removal of the drain due to respiratory failure and received a secondary drain during surgery. Surgical stabilization of the FC injuries was performed 7 days (IQR 2, 18) post admission, and the surgery lasted 132 (IQR 94, 200) minutes. We aimed to minimize surgical invasiveness while enhancing the thorax’s biomechanical stability by addressing the FC pathology. Therefore, we stabilized 3 (IQR 0, 3) and 2 (IQR 0, 3) ribs of the left and right hemithorax, respectively. This corresponds to 30% (IQR 10, 42.86) and 50% (IQR 29.29, 100) of the rib fractures and FC segments, respectively. Figure 2 depicts pre- and postoperative radiographs following surgical stabilization of an FC.

Over the documented follow-up period of 7 months (IQR 0, 48), 9.52% of the patients required surgical revision due to osteosynthetic failure. These were treated by implant removal. None of the patients had wound healing disorders following the initial or revision surgery.

There was complete documentation of the P_IP_, PEEP, FiO_2_, TV, RR, and AMV for 12, 12, 11, 6, 11, and 6 patients, respectively (Appendix A, Table A1).

### 3.2. Perioperative CT Lung Volume Changes

Perioperative lung volume changes were solely assessed in operatively treated hemithoraces. There were 17 hemithoraces with CT images before and after surgery. These scans were conducted 7 (IQR 2, 18) days before and 5 days (IQR 3, 10) following surgery. During the preoperative and postoperative scans, 61.9% and 60% of patients, respectively, were invasively ventilated. Correspondingly, there were no perioperative changes (*p* = 0.12) comparing the apex–diaphragm distance before (170 mm, IQR 153, 194) and after surgery (181 mm, IQR 162, 196).

The lung volumes of the surgically stabilized thorax side were 1185.8 cm^3^ (IQR 919.03, 1916.93) before and 1565.6 cm^3^ (IQR 1011.3, 1910.78) after surgery (*p* = 0.49).

The pooled mean lung volumes from the five imputation samples were 1476.08 cm^3^ (SEM 150.95) before surgery and 1452.83 cm^3^ (SEM 166.35) after surgery (*p* = 0.91).

## 4. Discussion

In this study, 21 patients were retrospectively assessed following a high-energy trauma. All patients had suffered an FC injury, which was operatively treated. The key finding of our study was that the surgical stabilization of the FC injury was not associated with early postoperative changes in the lung volumes measured in CT scans of the thorax compared to the preoperative volumes. Accordingly, early postoperative CT scans do not appear to be suitable for measuring the effectiveness of surgery on lung volumes.

Severe thoracic trauma leads to a significant increase in the necessary respiratory work in spontaneously breathing patients, which is associated with an increased risk for respiratory failure [54]. FC injuries are one of the most severe injuries following blunt thoracic trauma, as they are linked to a longer LOS and invasive ventilation duration compared to other blunt thoracic injuries [17,55]. Studies suggest the early surgical stabilization of FC injuries, as stabilization reduces the invasive ventilation duration and LOS and pneumonia risk [56,57]. Despite the outcome advantages following the osteosynthesis of FC injuries [58], fewer than one in five FC patients undergo surgery [31].

The primary rationale for the early stabilization of FC injuries is based on the enhanced biomechanical stability and restoration of the thoracic configuration following surgery. Few studies observed a significant increase in CT-measured lung volumes following the operative stabilization of FC injuries [46,47,48]. Caragounis et al. reported a cohort of 37 MT patients with FC injuries who underwent surgery using the MatrixRib^TM^ system [47]. Patients received a thorax CT of 0.6 mm thick slices at the three-month follow-up, which were manually and automatically measured using imaging software. Afterward, patients received an FVC and total lung capacity (TLC) measurement using body plethysmography. The authors observed a significant increase in the lung volumes of both thorax sides (left side from 1.46 to 2.69 L, right side from 1.9 to 2.94 L) and a significant correlation between the postoperative CT lung volumes and the FVC and TLC [47]. Büyükkarabacak et al. reported a cohort of 10 operatively and 10 non-operatively treated patients with an FC injury [48]. All patients received a CT thorax at the three-month follow-up, which showed that the volume increase comparing preoperative with postoperative CTs was higher in the operative group compared with the non-operative group. However, the authors did not statistically assess the pre- to postoperative volume changes. Further, the FVC, FEV1, and FEV/FVC ratios were not significantly different between the preoperative and postoperative measurements [48]. Last, Campbell et al. reported on 25 patients following the surgical stabilization of FC and non-flail rib fractures [46]. These patients had a follow-up period of 12 months, and lung volumes increased 1.71-fold in the FC and 1.69-fold in the non-FC fracture group compared to preoperatively [46]. The designs of these studies vary from those of our currently reported research.

Caragounis et al. reported a cohort highly comparable to ours [47]. Most patients were male, severely injured, and had an ISS of 20. They used the same osteosynthesis system we used in our study, stabilized an equal portion of FC segments, and measured lung volumes in a comparable manner [47]. Still, there is a main difference that may limit data comparability, as most of their patients spontaneously breathed during the CT scans. Only 4 out of 37 patients were invasively ventilated during the initial CT scan, whereas none of the patients were during the postoperative follow-up scan [47]. Due to the low number of patients with invasive ventilation, the authors could not report outcome measures for this subgroup compared to spontaneously breathing patients.

Büyükkarabacak et al. had, despite the thoracotomy for other reasons, comparable indications for surgery, but did not report the fraction of patients who were invasively ventilated during the CT scans [48]. This is highly relevant for data interpretation regarding volume measurement of the thorax in CT scans. Furthermore, the authors did not compare the actual preoperative and postoperative volumes in the operative group, which would have allowed for a direct conclusion regarding the primary impact of thoracic osteosynthesis. They compared all perioperative volumetric changes between the operatively and non-operatively treated thorax sides [48]. Lastly, they used manually shaped osteosyntheses, which differ from our data but are unlikely to have impacted the data of interest in the current context [48]. However, all previously mentioned studies compared a preoperative measurement with a measurement between 3 and 12 months postoperatively [46,47,48]. This long-term comparison varies significantly from our early postoperative assessments, complicating study comparability.

The evidence regarding the impact of operative FC stabilization on lung volumes in CT scans is limited. Evidence suggests improved lung function, especially the FVC [33,59,60], following the surgical stabilization of FC injuries. Besides clinical studies [33,61], this has already been shown in a human cadaver study, in which the loss of FC resulting from an FC injury is entirely restored following osteosynthesis of the flail segment [62]. However, previous studies reported only small cohorts, reducing the overall impact of the earlier results. Future studies should incorporate the standard lung function measurements and lung volume assessment into their design. Additionally, most studies have, thus far, excluded a relevant portion of MT patients, including patients with traumatic brain injury [32,33]. This has been a well-known problem in FC research.

The currently presented study has both strengths and limitations. First, this is the first study to assess volumetric changes in injured lungs in CT scans from primary hospital stays or early outpatient follow-ups. Previous studies focused on later follow-up, which our data add to. However, the absence of mid-term and long-term clinical and imaging outcome measures and outpatient follow-up limits our study’s informative value regarding the effects over time. This marks a significant limitation, as the lack of long-term follow-up data limits the overall relevance of our observations. We encourage future authors to conduct similar studies on a larger patient population with a more extended observation period.

Second, all patients have been treated according to the highest current treatment standard, as the primary study center is a level-one trauma center and is in charge of a large population area. Although the cohort is small, it is comparable to those reported in previous studies. A more extended recruitment period should be evaluated in future studies, or, as FC injuries after blunt trauma are rare, data collection should possibly be taken using a multicentric approach. Sufficiently large cohorts are needed for authors to make appropriate conclusions. The lack of significance in group differences in the current study may be partly related to the small sample size.

Further, most patients in the current study had been ventilated invasively during the CT scans. This may have impacted the volumetric measurements. As previously described [47], spontaneously breathing patients are the most optimal basis for lung volume measurements in CT. However, the cohort presented in this study is an MT cohort with severe injuries in various body regions following high-energy trauma. Therefore, invasive ventilation most commonly begins before hospital admission and, due to the high frequency of surgeries needed, does not end directly after hospital admission. We always aim to shorten invasive ventilation as much as possible. Still, most patients had not breathed spontaneously during the postoperative CT scans. Additionally, the inclusion of MT patients in the analysis could potentially impact the respiratory markers measured in our study, as MT patients follow different physiological needs compared to other patient cohorts. Although this was not the primary study outcome, this limitation should be considered when interpreting the data. Last, this retrospective study lacks a control group of conservatively treated FC patients for comparison. As a result, it is impossible to conclude the potential impact of surgery on the worsening of lung volumes when not performed.

## 5. Conclusions

This is the first study to retrospectively assess early postoperative outcomes in MT patients with FC injuries undergoing surgical fixation of the flail segment. Contrary to previous long-term observations, there were no pre- to postoperative changes in lung volumes when assessing short-term CT follow-up scans. CT-based lung volume changes may qualify as a surrogate parameter of surgical effectiveness following FC stabilization, but not in the short-term follow-up.

## Data Availability

Dataset available on request from the authors.

## Appendix A

**Table A1:** Respiratory markers.

	Last 72 h Preoperative	First 72 h Postoperative
P_IP_ [mbar]	20.39 (IQR 12.92, 24.36)	22.09 (IQR 13.4, 24.83)
PEEP [mbar]	9.67 (IQR 9.45, 9.99)	10.37 (IQR 8.79, 11.66)
FiO_2_ [%]	43.25 (IQR 37.25, 49.58)	45.38 (IQR 37.19, 50.6)
TV [mL]	461.04 (IQR 321.77, 600.73)	469.37 (IQR 317, 553.48)
RR [/min]	15.76 (IQR 13.41, 22.16)	17.34 (IQR 14.17, 18)
RMV [L/min]	8.49 (IQR 3.73, 9.2)	8.37 (IQR 5.04, 9.27)

Abbreviations: P_IP_: peak inspiratory pressure; PEEP: positive end-expiratory pressure; FiO_2_: fraction of inspired oxygen; TV: tidal volume; RR: respiratory rate; RMV: respiratory minute ventilation.
